# Overall and non‐lung cancer incidence and mortality in the National Lung Screening Trial: Opportunities for multi‐cancer early detection

**DOI:** 10.1002/cam4.7414

**Published:** 2024-06-22

**Authors:** Alpa V. Patel, Ellen T. Chang, Allan Hackshaw, Sam M. Janes, Diana S. M. Buist, Earl Hubbell, Christina A. Clarke, Graham A. Colditz

**Affiliations:** ^1^ American Cancer Society Atlanta Georgia USA; ^2^ GRAIL, LLC Menlo Park California USA; ^3^ Lung for Living, UCL Respiratory University College London London UK; ^4^ Department of Respiratory Medicine University College London London UK; ^5^ Washington University School of Medicine St. Louis Missouri USA

**Keywords:** cancer risk, cancer screening, early detection of cancer, multi‐cancer early detection, National Lung Screening Trial, population health

## Abstract

**Background:**

Currently recommended cancer screening programs address only part of the overall population cancer burden. Even populations deemed high‐risk for certain individual cancers experience a considerable potential burden of other cancers. However, few published cancer screening trials report the incidence of untargeted cancers.

**Methods:**

The National Lung Screening Trial (NLST), initiated in 2002–2004, was a randomized controlled trial of lung cancer screening in adults with ≥30 pack‐years of smoking. Active follow‐up for incident invasive cancers continued through 2009.

**Results:**

Among 53,229 NLST subjects (median follow‐up 6.5 years after randomization), the incidence of lung cancer was 615 per 100,000 person‐years (32% of 6142 overall first primary incident invasive cancers), and that of non‐lung cancer was 1327 per 100,000 (68%). Non‐lung cancer incidence exceeded that for lung cancer in all 5‐year age categories and all quintiles of smoking pack‐years. Besides lung cancer, the most common cancers were prostate, breast, colon/rectum, bladder, and head/neck; 23% were smoking‐related cancers, and 54% were cancer types lacking recommended population‐based screening modalities (32% excluding prostate). Non‐lung cancer comprised 48% of 1793 cancer deaths.

**Conclusions:**

In the NLST, only 32% of first primary cancer incidence after study entry was lung, compared with 68% non‐lung. Even in a population at high risk for lung cancer, a single‐cancer screening test misses most cancers. Thus, in combination with existing single‐cancer screening modalities, multi‐cancer screening tests—which address many of the incident non‐lung cancers in this trial—have potential to address a currently inaccessible portion of cancer morbidity and mortality.

## Graphical abstract

In the National Lung Screening Trial, lung cancer comprised less than one third of newly diagnosed cancers, whereas more than two thirds were non‐lung cancer types not targeted by the screening intervention. Even in this population at high risk for lung cancer, a single‐cancer screening test misses most cancers, which may instead be addressed by multi‐cancer early detection tests.

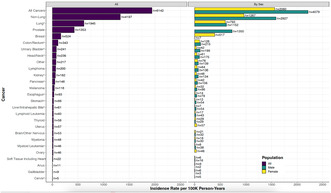

## 1 INTRODUCTION

Most public health goals for cancer control are based on the reduction of total population‐level cancer morbidity and mortality, yet prevention or early‐detection programs that target single cancer types are unlikely to substantially lessen the overall cancer burden. No single cancer type accounts for more than one fifth of cancer mortality (lung cancer comprises 21% of annual cancer deaths in the United States and 18% worldwide), and no single type accounts for more than one sixth of cancer incidence.^1^, ^2^ Therefore, single‐cancer screening strategies can address only a fraction of the total cancer burden, especially given incomplete adherence to screening recommendations.^3^ Strategies that simultaneously address multiple cancers have the potential to yield a far broader public health and economic benefit, especially as an addition to existing guideline‐based single‐cancer screening strategies.

The public health burden of cancers missed by single‐cancer screening strategies can be characterized by evaluating the burden of all cancer diagnoses and deaths in a population targeted for screening based on high risk for those cancer types. For example, lung cancer screening in the United States is targeted to persons designated as high‐risk based on age and smoking history. Older age and current smoking, in turn, are the two most important risk factors for the absolute and relative risks of all cancers combined.^4^ However, few cancer screening trials have reported the incidence of cancer types not targeted by the screening intervention (i.e., “untargeted” cancers). Although screening for invasive cancer cannot prevent cancer incidence (whereas screening for preinvasive lesions can be preventive), it can meaningfully affect outcomes by shifting diagnosis from later stages to earlier stages, when curative treatment is more likely to occur^5^ and quality of life may be improved.^6^ To further understand the overall cancer burden in a high‐risk population targeted for lung cancer screening, we evaluated the incidence and mortality of all invasive cancers, especially non‐lung, in the National Lung Screening Trial (NLST; NCT00047385), a US multicenter, randomized controlled trial comparing up to three rounds of screening of either low‐dose helical computed tomography (CT) or chest radiography for lung cancer screening among current and former heavy smokers.^7^, ^8^

## 2 METHODS

The NLST study design has previously been described in detail.^7^, ^8^ Briefly, between August 2002 and April 2004, 53,452 adults aged 55–74 years at randomization, who had no history of cancer in the previous 5 years, had a ≥30‐pack‐year history of cigarette smoking, and, if former smokers, had quit within the previous 15 years, were enrolled from 33 participating US medical institutions. Individuals were excluded if they had a past diagnosis of lung cancer, chest CT within 18 months before enrollment, hemoptysis, or unexplained weight loss of >6.8 kg in the preceding year. Participants were randomized to three rounds of screening with either low‐dose CT or chest radiography, at baseline and two annual follow‐up examinations. The NLST was approved by the institutional review boards at the 33 participating medical institutions, and all participants provided written informed consent before randomization.

Participants were actively followed for all newly diagnosed cancers and vital status from study randomization through December 31, 2009. Incident cancers and deaths were identified by annual or semiannual participant‐completed questionnaires, death certificates obtained through linkage with the National Death Index, and, rarely, direct notification by a participant's relative; all positive notifications of an incident cancer diagnosis were confirmed by medical record abstraction.^9^ Information was not collected on route to diagnosis (e.g., screening, general practitioner referral, emergency presentation) of non‐lung cancers, or use of any cancer screening besides study‐assigned lung cancer screening. Data on all incident cancers diagnosed during follow‐up (up to four cancers per person) were coded using International Classification of Diseases for Oncology, 3rd edition (ICD‐O‐3) codes for topography, morphology, behavior, and grade, as well as Surveillance, Epidemiology, and End Results (SEER) site categorizations derived from the ICD‐O‐3 codes. The present analysis includes first invasive primary cancers (ICD‐O‐3 behavior code 3/malignant) diagnosed after study randomization.

Death certificates were obtained for participants who were known to have died, and were used to classify cause of death. We included cancer deaths even if they were not preceded by a recorded incident cancer diagnosis, since cancer incidence may have been under‐ascertained based on participant‐completed questionnaires.

As previously reported,^7^, ^8^ lung cancer incidence was modestly higher in the low‐dose CT arm compared with the chest radiography arm during the active follow‐up period (median 6.5 years), but this difference disappeared after extended follow‐up (median 11.3 years), and non‐lung cancer incidence was similar between the two study arms; therefore, we combined the two arms for analysis. Lung cancer mortality as of 2009 was reduced by 20% in the low‐dose CT arm versus the chest radiography arm, but the percentage of deaths from other cancers was similar between arms (22.3% and 22.2% of all deaths, respectively).^7^ Incidence rates were calculated using person‐time from randomization until cancer diagnosis, loss to follow‐up (<4% of the study population), or the end of 2009, whichever occurred first. Mortality rates were calculated per 100,000 persons through the end of 2009.

After excluding 225 subjects marked as ineligible or with missing or implausible values for dates or tumor type (Table S1), 53,229 eligible participants remained for analysis. We examined the following outcomes: incidence and mortality of all cancers; lung and non‐lung cancers; cancers lacking currently recommended population screening guidelines based on United States Preventive Services Task Force (USPSTF) A‐ and B‐grade recommendations (i.e., types other than lung, female breast, colon/rectum, and cervix),^10^ and unscreened cancer types after additionally excluding prostate, which is not recommended by USPSTF (C‐grade); smoking‐related cancers besides lung (i.e., colon/rectum, bladder, head/neck, kidney, pancreas, esophagus, stomach, liver, acute myeloid leukemia, and cervix)^11^, ^12^; and uncommon cancers, including rare cancers defined by the National Cancer Institute as those affecting <40,000 persons per year in the United States,^13^ that is, myeloma, stomach cancer, brain/other nervous system cancer, esophageal cancer, specific head and neck cancers, leukemia types, and other, lower‐incidence cancers.^2^

For comparisons of cancer incidence between the NLST study population and the general US population, we used crude incidence rates of first primary invasive cancers from 2002 to 2009 among US adults aged 55–79 years in 22 SEER geographic regions,^14^ accessed using SEER*Stat software.^15^

## 3 RESULTS

**FIGURE 1:**
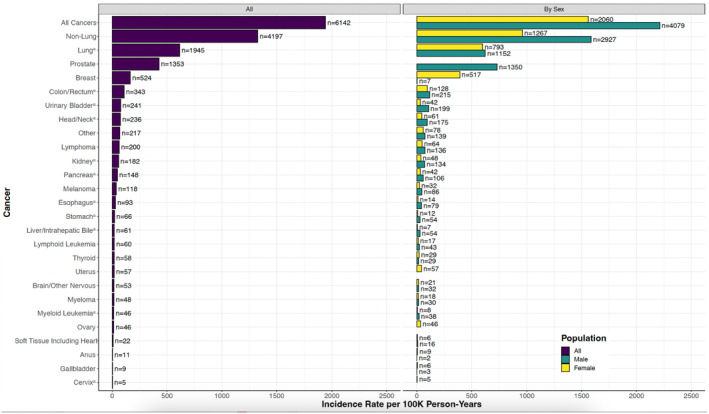
Incidence rates (per 100,000 person‐years) of first primary invasive cancers diagnosed during follow‐up after randomization in the National Lung Screening Trial (NLST) study population by cancer type and sex. ^a^Smoking‐related cancers.^11^, ^12^

Based on 6142 first primary invasive cancers diagnosed during active follow‐up of this population at high risk for lung cancer, the overall incidence rate of invasive cancer after study entry was 1942 per 100,000 person‐years; 32% of these cancers were lung cancer (615 per 100,000 person‐years, *n* = 1945), while 68% were not lung cancer (1327 per 100,000 person‐years, *n* = 4197). Of the incident lung cancer cases, 901 (46%) were associated with a positive low‐dose CT or chest radiography screening test; 793 (41%) occurred after the study screening period. Route to diagnosis (screening‐related or not) was not reported for cancer types other than lung. Cancer case counts and incidence rates are summarized in Figure 1 and Table 1. No currently recommended population‐based screening strategy exists for 54% (3332 of 6142) of the total observed incident cancer cases (32% after additionally excluding prostate cancer; 1979 of 6142). Smoking‐related cancers other than lung comprised 23% (1421 of 6142), while non‐smoking‐related cancers comprised 45% (2776 of 6142). Individually uncommon cancer types collectively accounted for 16% of observed cancer cases (969 of 6142).

**TABLE 1:** Case counts and incidence rates (per 100,000 person‐years) of first primary invasive cancer diagnosed during follow‐up after randomization in the National Lung Screening Trial (NLST) study population.

Primary cancer type	Total (*n* = 53,229)		Men (*n* = 31,411)		Women (*n* = 21,818)	
	Cases (*n*)	Incidence rate	Cases (*n*)	Incidence rate	Cases (*n*)	Incidence rate
Total	6142	1942	4079^a^	2212	2060	1561
Non‐lung	4197	1327	2927^a^	1587	1267	960
Lung^b^	1945	615	1152	625	793	601
Prostate	1353	428	1350^a^	732	n/a	n/a
Breast	524	166	7	4	517	392
Colon/rectum^b^	343	108	215	117	128	97
Urinary bladder^b^	241	76	199	108	42	32
Head/neck^b^	236	75	175	95	61	46
Other	217	69	139	75	78	59
Lymphoma	200	63	136	74	64	49
Kidney^b^	182	58	134	73	48	36
Pancreas^b^	148	47	106	57	42	32
Melanoma	118	37	86	47	32	24
Esophagus^b^	93	29	79	43	14	11
Stomach^b^	66	21	54	29	12	9
Liver/intrahepatic bile duct^b^	61	19	54	29	7	5
Lymphoid leukemia	60	19	43	23	17	13
Thyroid	58	18	29	16	29	22
Uterus	57	18	n/a	n/a	57	43
Brain/other nervous system	53	17	32	17	21	16
Myeloma	48	15	30	16	18	14
Myeloid leukemia^b^	46	15	38	21	8	6
Ovary	46	15	n/a	n/a	46	35
Soft tissue Including heart	22	7	16	9	6	5
Anus	11	3	2	1	9	7
Gallbladder	9	3	3	2	6	5
Cervix^b^	5	2	n/a	n/a	5	4

Abbreviation: n/a, not applicable.

^a^ Excludes three cases with prostate cancer coded as female.

^b^ Smoking‐related cancers.^11^, ^12^

**FIGURE 2:**
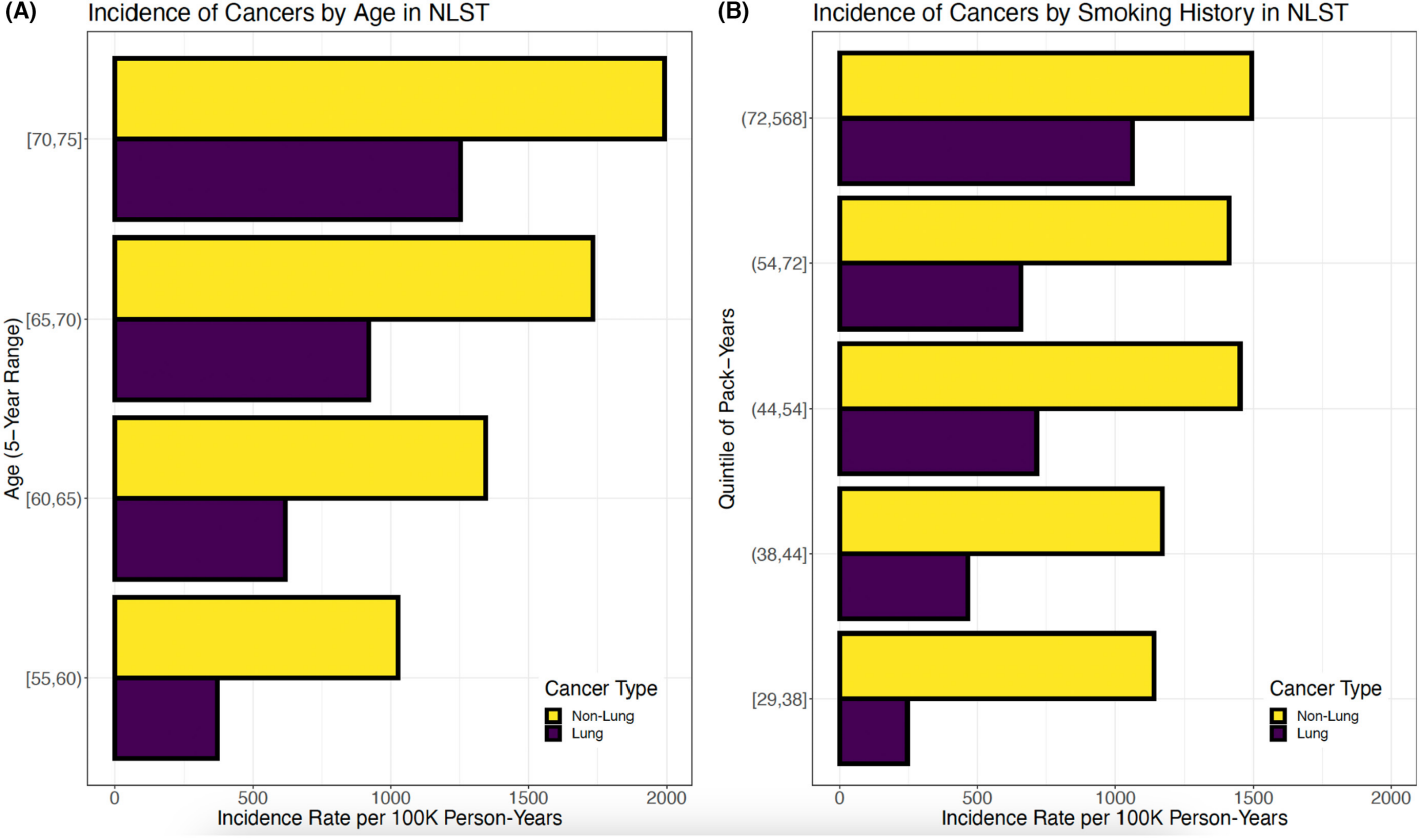
Incidence rates (per 100,000 person‐years) of first primary invasive lung and non‐lung cancers diagnosed during follow‐up after randomization in the National Lung Screening Trial (NLST) study population stratified by (A) age at enrollment or (B) cigarette smoking pack‐years at enrollment.

In all 5‐year categories of age at enrollment, the non‐lung cancer incidence rate exceeded that for lung cancer, especially at younger ages (Figure 2A). Non‐lung cancer also exceeded lung cancer incidence in all quintiles of smoking pack‐years at enrollment, especially in persons with fewer pack‐years (Figure 2B). An alternative presentation of these results with cancer types classified as lung, other smoking‐related (besides lung), and non‐smoking‐related cancers is provided in Figure S1, which shows that the incidence of non‐smoking‐related cancers exceeded that of lung cancer in all age groups and all except the highest quintile of smoking pack‐years.

The overall cancer incidence rate after study entry in the NLST (incidence rates per 100,000 person‐years: 1942 overall, 2212 in men, 1561 in women) exceeded that in the general US SEER population of adults aged 55–79 years from 2002 to 2009 (1543 overall, 1877 in men, 1252 in women) (Table 2; Figure S2). Most of the excess was due to lung cancer; otherwise, the incidence rate of non‐lung cancers was comparable between NLST participants (1327 overall, 1587 in men, and 960 in women) and the general SEER population (1306 overall, 1598 in men, and 1052 in women), with excesses of several strongly smoking‐related cancers (e.g., head/neck and esophagus) balanced by deficits of other cancers (e.g., colon/rectum and melanoma).

**TABLE 2:** Primary invasive cancer incidence rates (per 100,000 person‐years) in the National Lung Screening Trial (NLST) study population (enrolled in 2002–2004, followed through 2009) and among US adults aged 55–79 years in the Surveillance, Epidemiology, and End Results (SEER) 22 registries, 2002–2009.

	NLST			SEER		
	Total (*n*)	Men (*n*)	Women (*n*)	Total (*n*)	Men (*n*)	Women (*n*)
Total	1942	2212	1561	1543	1877	1252
Non‐lung	1327	1587	960	1306	1598	1052
Lung^a^	615	625	601	237	280	201
Prostate	428	732	n/a	299	642	n/a
Breast	166	4	392	195	4	361
Colon/rectum^a^	108	117	97	150	178	127
Urinary bladder^a^	76	108	32	71	117	31
Head/neck^a^	75	95	46	50	79	25
Other	69	75	59	96	109	85
Lymphoma	63	74	49	62	72	53
Kidney^a^	58	73	36	50	69	34
Pancreas^a^	47	57	32	39	44	35
Melanoma	37	47	24	49	69	32
Esophagus^a^	29	43	11	17	29	7
Stomach^a^	21	29	9	24	33	16
Liver/intrahepatic bile duct^a^	19	29	5	24	38	13
Lymphoid leukemia	19	23	13	19	26	14
Thyroid	18	16	22	21	14	27
Uterus	18	n/a	43	45	n/a	84
Brain/other nervous system	17	17	16	15	19	13
Myeloma	15	16	14	21	25	18
Myeloid leukemia^a^	15	21	6	15	18	12
Ovary	15	n/a	35	20	n/a	38
Soft tissue including heart	7	9	5	7	9	6
Anus	3	1	7	5	4	5
Gallbladder	3	2	5	4	3	5
Cervix^a^	2	n/a	4	7	n/a	14

Abbreviation: n/a, not applicable.

^a^ Smoking‐related cancers.^11^, ^12^

Parallel results for cancer mortality in the NLST study population are presented in Table 3 and illustrated in the Data S1. Of the 1793 cancer deaths that occurred through 2009, 933 (52%; 1753 per 100,000 persons) were from lung cancer and 860 (48%; 1616 per 100,000 persons) were from cancer types other than lung (Table 3; Figure S3). The proportion of cancer mortality from non‐lung types was higher among men (50%; 1891 per 100,000 persons, *n* = 594) than women (44%; 1219 per 100,000 persons, *n* = 266). Of all cancer deaths, 42% (757 of 1793) were from types lacking currently recommended screening guidelines (41% after additionally excluding prostate cancer; 732 of 1793). Smoking‐related cancers other than lung represented a higher percentage of cancer deaths than incidence, accounting for 30% of cancer deaths (536 of 1793), while non‐smoking‐related cancers accounted for 18% (324 of 1793). Individually uncommon cancer types comprised 23% of cancer deaths (419 of 1793). Mortality rates from lung and non‐lung cancers were comparable across categories of age at enrollment up to 69 years, but lung cancer mortality was appreciably higher in the oldest group (ages 70–75 years at enrollment) (Figure S4A). Similarly, mortality from lung and non‐lung cancers was similar across most quintiles of smoking pack‐years at study entry, except in the highest quintile (Figure S4B). Mortality from smoking‐related cancers other than lung exceeded that from non‐smoking‐related cancers in all categories (Figures S5A,B).

**TABLE 3:** Death counts and cancer‐specific mortality rates (per 100,000 persons) during follow‐up after randomization in the National Lung Screening Trial (NLST) study population.

Primary cancer type	Total (*n* = 53,229)		Men (*n* = 31,411)		Women (*n* = 21,818)	
	Deaths (*n*)	Mortality rate	Deaths (*n*)	Mortality rate	Deaths (*n*)	Mortality rate
Total	1793	3368	1189	3785	604	2768
Non‐lung	860	1616	594	1891	266	1219
Lung^a^	933	1753	595	1894	338	1549
Pancreas^a^	160	301	113	360	47	215
Other	106	199	77	245	29	133
Esophagus^a^	73	137	64	204	9	41
Colon/rectum^a^	69	130	41	131	28	128
Brain/other nervous system	53	100	32	102	21	96
Myeloid leukemia^a^	53	100	41	131	12	55
Liver/intrahepatic bile duct^a^	47	88	39	124	8	37
Head/neck^a^	45	85	35	111	10	46
Urinary bladder^a^	44	83	37	118	7	32
Breast	33	62	0	0	33	151
Lymphoma	30	56	23	73	7	32
Prostate	25	47	25	80	n/a	n/a
Ovary	23	43	n/a	n/a	23	105
Stomach^a^	23	43	18	57	5	23
Kidney^a^	21	39	16	51	5	23
Myeloma	20	38	12	38	8	37
Soft tissue including heart	9	17	8	25	1	5
Melanoma	8	15	6	19	2	9
Lymphoid leukemia	4	8	4	13	0	0
Thyroid	4	8	1	3	3	14
Uterus	4	8	n/a	n/a	4	18
Gallbladder	3	6	1	3	2	9
Anus	2	4	1	3	1	5
Cervix^a^	1	2	n/a	n/a	1	5

Abbreviation: n/a, not applicable.

^a^ Smoking‐related cancers.^11^, ^12^

## 4 DISCUSSION

In the NLST, a population designated as high‐risk for targeted lung cancer screening, only 32% of first primary cancer incidence was lung; non‐lung cancer comprised more than twice as many cancers (68% of total cancers) after study entry, and more than half (54%) of incident cancers were types that currently have no generally recommended population‐based screening strategy. Nearly half of cancer deaths (48%) were from non‐lung cancer (absolute mortality rate = 1.6%), including 42% without recommended screening guidelines. The most common incident cancer types after lung were the same as in the general US population^2^ (prostate, breast, and colon/rectum), as well as other smoking‐related cancers such as urinary bladder and head/neck.^11^, ^12^ These findings illustrate that even in a high‐risk population—here, enriched for lung cancer by age and extensive smoking history, the two leading risk factors contributing to absolute risk of all cancers combined^4^—a single‐cancer screening test omits the majority of cancers diagnosed in the screened population. These results also highlight the value of smoking cessation for preventing multiple cancers in addition to lung.

Multi‐cancer early detection (MCED) tests can address a broad spectrum of cancers, including common and uncommon cancers that do not have recommended population‐based screening strategies, giving such tests the potential to make a substantial impact on reducing cancer morbidity and mortality as an adjunct to existing guideline‐based screening. MCED tests are designed to maintain a very low overall false‐positive rate for all cancers combined, for example, 0.5% in a large validation study of one commercially available MCED test,^16^ Galleri®, that is currently being evaluated for clinical utility in a large, randomized controlled trial.^17^ In contrast, combined single‐cancer screening tests, which generally have higher sensitivity for individually targeted cancer types than MCED tests, have a high cumulative false‐positive rate that is two orders of magnitude higher (e.g., 49% for women and 60% for men at the end of the 3‐year screening period in the Prostate, Lung, Colorectal, and Ovarian [PLCO] Cancer Screening Trial).^18^ Thus, MCED tests can be used in addition to existing screening without adding a large false‐positive burden.^19^ Other issues that merit further consideration in the development and implementation of MCED tests include variation in tumor biology, clinical course, risk factors, treatment options, and other disease characteristics, many of which have been evaluated in clinical research and modeling studies.^16^, ^17^, ^19^, ^20^, ^21^, ^22^, ^23^, ^24^

This study is strengthened by its use of a well‐described screening population for whom elevated risk was clearly defined, with high completeness of follow‐up (96% for vital status^7^). A minor limitation is the ascertainment of incident cancers based largely on self‐reported questionnaire data, although positive reports were validated by medical records review. Thus, overall cancer incidence, especially for non‐lung cancer, may have been underestimated in both trial arms, although probably not substantially so, given the trial setting. We did not distinguish screen‐detected from non‐screen‐detected lung cancers, or compare their mortality with that expected in the general population. Additionally, for non‐lung cancers, the NLST did not collect information on stage at diagnosis, use of screening, results of any screening tests (including false positives), or route to diagnosis.

Evaluating the incidence and mortality of all cancers in study populations targeted by single‐cancer or selected‐cancer screening strategies helps contextualize the proportion of the cancer burden addressed by the intervention. Although numbers are often unreported, the proportion of untargeted cancers, when reported, is substantial. For example, in the PLCO trial of 154,901 men and women aged 55–74 years, targeted cancers comprised 55% of incident cancers identified during 13 years of follow‐up (*n* = 14,698, including 8468 prostate, 3567 lung, 2291 colon/rectum, and 372 ovary), whereas cancers untargeted by the screening intervention comprised 45% (*n* = 11,850, including 4438 breast, 2555 hematologic, 1430 bladder, 776 kidney, and 753 pancreas).^25^ In the Cancer of Pancreas Screening‐5 trial of 1461 high‐risk individuals with germline susceptibility gene variants and/or family history of pancreatic cancer, nine patients were diagnosed with pancreatic cancer during study surveillance, whereas 73 other, non‐pancreatic cancers were diagnosed during the same time frame^26^; thus, the screened cancer comprised 11% of total cancer incidence in a cohort enriched for pancreatic cancer. Although few large cancer screening trials have reported the incidence of untargeted cancers or other competing risks, several reported that mortality from untargeted cancers exceeded that of targeted types, such as lung, breast, prostate, colorectal, and ovarian cancers.^27^, ^28^, ^29^, ^30^, ^31^, ^32^, ^33^ Most of the untargeted cancer types in these trials can be detected by MCED tests, although a mortality benefit of MCED screening has not yet been demonstrated.

## 5 CONCLUSION

To our knowledge, no major cancer screening trials have reported the incidence of untargeted cancers by stage at diagnosis, which would enable estimation of the potential burden of cancer averted through targeted or incidental early detection. Yet, cancer screening trials often report all‐cancer and all‐cause mortality as secondary study endpoints. Even with cancer likely to surpass cardiovascular disease globally as the leading cause of premature death,^34^ single‐cancer screening strategies address at most only one fifth of total cancer morbidity and mortality^1^, ^2^ and a considerably smaller fraction of all‐cause mortality. Therefore, we encourage screening trial investigators to routinely report the incidence of all cancer types, ideally by stage at diagnosis, and cancer mortality by type when publishing trial results. In screening trials for selected high‐risk populations, such results can identify other cancer types that might be influenced by cancer control measures (e.g., screening of genetically susceptible groups). We also urge reporting of all cancer screening activities, including results, within screening trial populations to enable evaluation of the total false‐positive burden, adherence to multiple screening regimens, and other endpoints.^19^ More broadly, reporting of overall cancer incidence and mortality data provide useful insight on how far we still have to go to reduce the population burden of cancer, and—in the future—such data can shed light on how far we have progressed toward achieving this overarching goal. To maximize the reduction of morbidity and mortality from any cancer, strategies such as MCED tests should be considered in combination with existing recommended single‐cancer screening modalities.

## AUTHOR CONTRIBUTIONS

**Alpa V. Patel:** Investigation (supporting); writing – original draft (supporting); writing – review and editing (supporting). **Ellen T. Chang:** Conceptualization (lead); investigation (supporting); methodology (supporting); writing – original draft (lead); writing – review and editing (lead). **Allan Hackshaw:** Writing – review and editing (supporting). **Sam M. Janes:** Writing – review and editing (supporting). **Diana S. M. Buist:** Conceptualization (supporting); investigation (supporting); writing – original draft (supporting); writing – review and editing (supporting). **Earl Hubbell:** Conceptualization (supporting); formal analysis (lead); investigation (supporting); methodology (lead); writing – original draft (supporting); writing – review and editing (supporting). **Christina A. Clarke:** Conceptualization (supporting); investigation (supporting); methodology (supporting); writing – original draft (supporting); writing – review and editing (supporting). **Graham A. Colditz:** Investigation (supporting); writing – original draft (supporting); writing – review and editing (supporting).

## DISCLOSURES

AVP and GAC report no relevant conflicts of interest. ETC, DSMB, EAH, and CAC are employees of GRAIL, LLC, report stock or other support from Illumina, and report other support from GRAIL, LLC, during the conduct of the study. In addition, EAH has multiple patents in the field of cancer detection pending to GRAIL, LLC. AH reports an honorarium for an advisory board meeting for GRAIL, LLC; a consultation fee for Evidera for a GRAIL‐initiated project; and previously owned shares in Illumina. SMJ reports a research grant awarded from GRAIL, LLC, for a separate study; honoraria for travel, consultancy, or speaking from AstraZeneca, BARD1 Bioscience, Optellum, Janssen, Takeda, Evidera, and Achilles Therapeutics; grant funding from Owlstone for a separate research study; and a family member who is an employee of AstraZeneca.

## Supporting information


Data S1:

## Data Availability

The authors thank the National Cancer Institute (NCI) for access to NCI's data collected by the NLST, granted for CDAS project number NLST‐995. NLST has the ClinicalTrials.gov number NCT00047385.

## References

[1] Ferlay J , Ervik M , Lam F , et al. Global Cancer Observatory: Cancer Today. 2020 Accessed 17 January 2023. http://gco.iarc.fr/today/home

[2] American Cancer Society . Cancer Facts & Figures 2024. American Cancer Society; 2024. https://www.cancer.org/research/cancer‐facts‐statistics/all‐cancer‐facts‐figures/2024‐cancer‐facts‐figures.html

[3] Philipson TJ , Durie T , Cong Z , Fendrick AM . The aggregate value of cancer screenings in the United States: full potential value and value considering adherence. BMC Health Serv Res. 2023;23:829. doi:10.1186/s12913-023-09738-4 37550686 PMC10405449

[4] Patel AV , Deubler E , Teras LR , et al. Key risk factors for the relative and absolute 5‐year risk of cancer to enhance cancer screening and prevention. Cancer. 2022;128(19):3502‐3515. doi:10.1002/cncr.34396 35920750 PMC9544865

[5] Hubbell E , Clarke CA , Smedby KE , Adami HO , Chang ET . Potential for cure by stage across the cancer spectrum in the United States. Cancer Epidemiol Biomarkers Prev. 2023;33(2):206‐214. doi:10.1158/1055-9965.EPI-23-1018 PMC10844847 38019271

[6] Neal RD , Tharmanathan P , France B , et al. Is increased time to diagnosis and treatment in symptomatic cancer associated with poorer outcomes? Systematic review. Br J Cancer. 2015;112(Suppl 1):S92‐S107. doi:10.1038/bjc.2015.48 25734382 PMC4385982

[7] The National Lung Screening Trial Research Team . Reduced lung‐cancer mortality with low‐dose computed tomographic screening. N Engl J Med. 2011;365(5):395‐409. doi:10.1056/NEJMoa1102873 21714641 PMC4356534

[8] The National Lung Screening Trial Research Team . Lung cancer incidence and mortality with extended follow‐up in the National Lung Screening Trial. J Thorac Oncol. 2019;14(10):1732‐1742. doi:10.1016/j.jtho.2019.05.044 31260833 PMC6764895

[9] National Cancer Institute . *Other (Non‐Lung) Cancer‐Cancer Diagnosis–Learn–NLST–The Cancer Data Access System*, *Learn about NLST: Cancer Diagnosis: Other (Non‐Lung) Cancer* . 2022 Accessed: 20 January 2023. https://cdas.cancer.gov/learn/nlst/cancer‐dx/non‐lung/

[10] US Preventive Services Task Force . A and B Recommendations . 2023. Accessed: 16 May 2023. https://www.uspreventiveservicestaskforce.org/uspstf/recommendation‐topics/uspstf‐a‐and‐b‐recommendations

[11] US Surgeon General . The health consequences of smoking‐‐50 years of progress: a report of the Surgeon General. Public Health Service, Office of the Surgeon General. US Department of Health and Human Services; 2014. doi:10.1037/e510072014-001

[12] Centers for Disease Control and Prevention . *Smoking and Cancer*, *The Tips From Former Smokers campaign features real people suffering as a result of smoking and exposure to secondhand smoke* . 2022 Accessed: 20 January 2023. https://www.cdc.gov/tobacco/campaign/tips/diseases/cancer.html

[13] National Cancer Institute . About Rare Cancers–NCI . 2019 Accessed: 27 February 2023. https://www.cancer.gov/pediatric‐adult‐rare‐tumor/rare‐tumors/about‐rare‐cancers

[14] SEER . Surveillance, Epidemiology, and End Results (SEER) Program (www.seer.cancer.gov) SEER*Stat Database: Incidence–SEER Research Limited‐Field Data, 22 Registries, Nov 2022 Sub (2000‐2020) –Linked to County Attributes–Time Dependent (1990‐2021) Income/Rurality, 1969‐2021 Counties. National Cancer Institute, DCCPS, Surveillance Research Program; 2023.

[15] Surveillance Research Program, National Cancer Institute . SEER*Stat software (seer.cancer.gov/seerstat) version 8.4.1. 2023.

[16] Klein EA , Richards D , Cohn A , et al. Clinical validation of a targeted methylation‐based multi‐cancer early detection test using an independent validation set. Ann Oncol. 2021;32(9):1167‐1177. doi:10.1016/j.annonc.2021.05.806 34176681

[17] Neal RD , Johnson P , Clarke CA , et al. Cell‐free DNA‐based multi‐cancer early detection test in an asymptomatic screening population (NHS‐Galleri): design of a pragmatic, prospective randomised controlled trial. Cancer. 2022;14(19):4818. https://www.mdpi.com/2072‐6694/14/19/4818 10.3390/cancers14194818 PMC9564213 36230741

[18] Croswell JM , Kramer BS , Kreimer AR , et al. Cumulative incidence of false‐positive results in repeated, multimodal cancer screening. Ann Fam Med. 2009;7(3):212‐222. doi:10.1370/afm.942 19433838 PMC2682972

[19] Hackshaw A , Cohen SS , Reichert H , Kansal AR , Chung KC , Ofman JJ . Estimating the population health impact of a multi‐cancer early detection genomic blood test to complement existing screening in the US and UK. Br J Cancer. 2021;125(10):1432‐1442. doi:10.1038/s41416-021-01498-4 34426664 PMC8575970

[20] Schrag D , Beer TM , McDonnell CH III , et al. Blood‐based tests for multicancer early detection (PATHFINDER): a prospective cohort study. Lancet. 2023;402(10409):1251‐1260. doi:10.1016/S0140-6736(23)01700-2 37805216 PMC11027492

[21] Hubbell E , Clarke CA , Aravanis AM , Berg CD . Modeled reductions in late‐stage cancer with a multi‐cancer early detection test. Cancer Epidemiol Biomarkers Prev. 2021;30(3):460‐468. doi:10.1158/1055-9965.EPI-20-1134 33328254

[22] Dai J , Zhang J , Braun JV , Simon N , Hubbell E , Zhang N . Clinical performance and utility: a microsimulation model to inform the design of screening trials for a multi‐cancer early detection test. J Med Screen, Published online February 2, 2024. 2024. doi:10.1177/09691413241228041 PMC11330083 38304990

[23] Etzioni R , Gulati R , Patriotis C , et al. Revisiting the standard blueprint for biomarker development to address emerging cancer early detection technologies. J Natl Cancer Inst. 2024;116(2):189‐193. https://academic.oup.com/jnci/article/116/2/189/7371303 37941446 10.1093/jnci/djad227 PMC10852609

[24] Lange J , Gogebakan KC , Gulati R , Etzioni R . Projecting the impact of multi‐cancer early detection on late‐stage incidence using multi‐state disease modeling. Cancer Epidemiol Biomarkers Prev, Published online April 11, 2024. 2024;33:830‐837. doi:10.1158/1055-9965.EPI-23-1470 38506751 PMC11213491

[25] Zhu CS , Pinsky PF , Kramer BS , et al. The prostate, lung, colorectal, and ovarian cancer screening trial and its associated research resource. J Natl Cancer Inst. 2013;105(22):1684‐1693. doi:10.1093/jnci/djt281 24115361 PMC3888207

[26] Dbouk M , Katona BW , Brand RE , et al. The multicenter cancer of pancreas screening study: impact on stage and survival. J Clin Oncol. 2022;40(28):3257‐3266. doi:10.1200/JCO.22.00298 35704792 PMC9553376

[27] de Koning HJ , van der Aalst CM , de Jong PA , et al. Reduced lung‐cancer mortality with volume CT screening in a randomized trial. N Engl J Med. 2020;382(6):503‐513. doi:10.1056/NEJMoa1911793 31995683

[28] Wille MMW , Dirksen A , Ashraf H , et al. Results of the randomized Danish lung cancer screening trial with focus on high‐risk profiling. Am J Respir Crit Care Med. 2016;193(5):542‐551. doi:10.1164/rccm.201505-1040OC 26485620

[29] Jacobs IJ , Menon U , Ryan A , et al. Ovarian cancer screening and mortality in the UK collaborative trial of ovarian cancer screening (UKCTOCS): a randomised controlled trial. Lancet. 2016;387(10022):945‐956. doi:10.1016/S0140-6736(15)01224-6 26707054 PMC4779792

[30] Friedman DR , Dubin N . Case‐control evaluation of breast cancer screening efficacy. Am J Epidemiol. 1991;133(10):974‐984. doi:10.1093/oxfordjournals.aje.a115817 2035508

[31] Andersson I , Aspegren K , Janzon L , et al. Mammographic screening and mortality from breast cancer: the Malmö mammographic screening trial. BMJ. 1988;297(6654):943‐948. 3142562 10.1136/bmj.297.6654.943 PMC1834636

[32] Pinsky PF , Miller E , Prorok P , Grubb R , Crawford ED , Andriole G . Extended follow‐up for prostate cancer incidence and mortality among participants in the prostate, lung, colorectal and ovarian randomized cancer screening trial. BJU Int. 2019;123(5):854‐860. doi:10.1111/bju.14580 30288918 PMC6450783

[33] Kronborg O , Jørgensen OD , Fenger C , Rasmussen M . Randomized study of biennial screening with a faecal occult blood test: results after nine screening rounds. Scand J Gastroenterol. 2004;39(9):846‐851. doi:10.1080/00365520410003182 15513382

[34] Bray F , Laversanne M , Weiderpass E , Soerjomataram I . The ever‐increasing importance of cancer as a leading cause of premature death worldwide. Cancer. 2021;127(16):3029‐3030. doi:10.1002/cncr.33587 34086348

