# Supplementary material for: Overall and non‐lung cancer incidence and mortality in the National Lung Screening Trial: Opportunities for multi‐cancer early detection
Source: Cancer Med. 2024 Jun 22;13(12):e7414. doi: 10.1002/cam4.7414 (PMC11192995; doi:10.1002/cam4.7414)
Supplement: Supplementary file 1 — Data S1: [file CAM4-13-e7414-s001.docx]

**Data S1**

**Figure S1. Incidence rates (per 100,000 person-years) of first primary invasive lung, other smoking-related, and non-smoking-related cancers diagnosed during follow-up after randomization in the National Lung Screening Trial (NLST) study population stratified by A) age at enrollment or B) cigarette smoking pack-years at enrollment.**

**
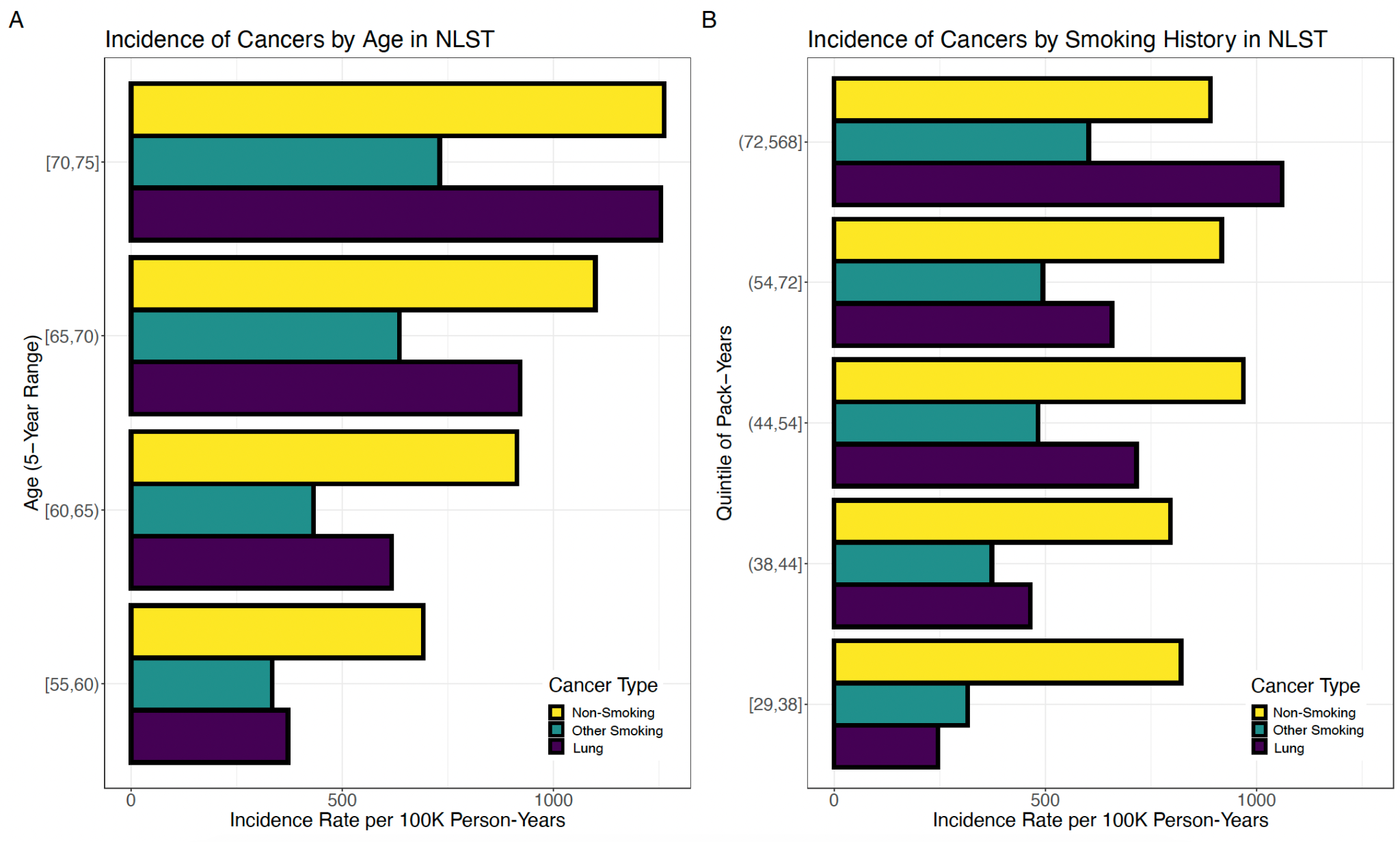
**

**Figure S2. Incidence rates (per 100,000 person-years) of first primary invasive cancers diagnosed during follow-up after randomization in the National Lung Screening Trial (NLST) study population by cancer type and sex, compared with incidence rates among US adults aged 55–79 years in the Surveillance, Epidemiology, and End Results (SEER) 22 registries, 2002–2009. SEER incidence rates are indicated by blue dots. ^a^Smoking-related cancers.^11,12^**

**
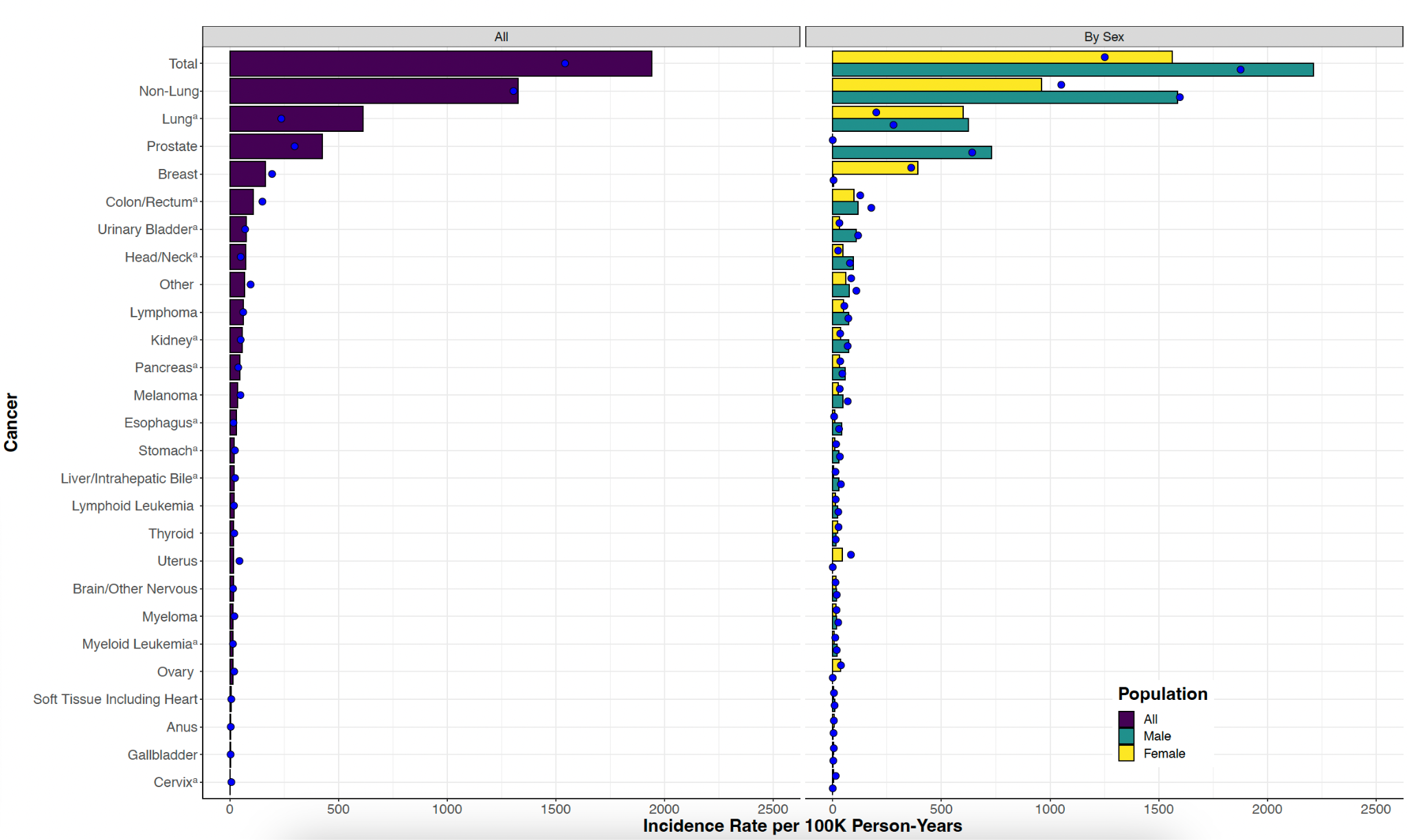
**

**Figure S3. Cancer-specific mortality rates (per 100,000 persons) during follow-up after randomization in the National Lung Screening Trial (NLST) study population by cancer type and sex. ^a^Smoking-related cancers.^11,12^**

**
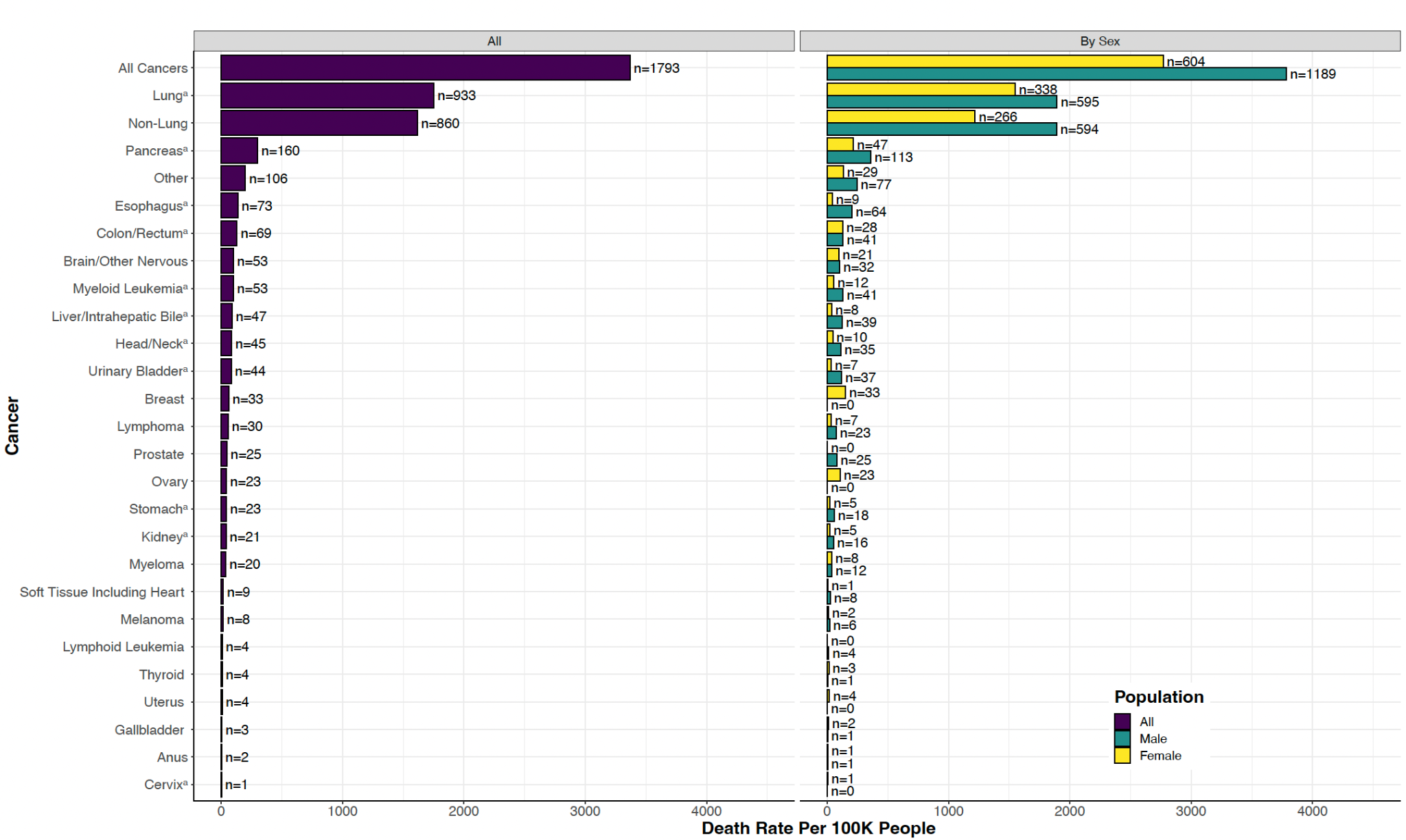
**

**Figure S4. Cancer-specific mortality rates (per 100,000 persons) from lung and non-lung cancers during follow-up after randomization in the National Lung Screening Trial (NLST) study population stratified by A) age at enrollment or B) cigarette smoking pack-years at enrollment.**

**
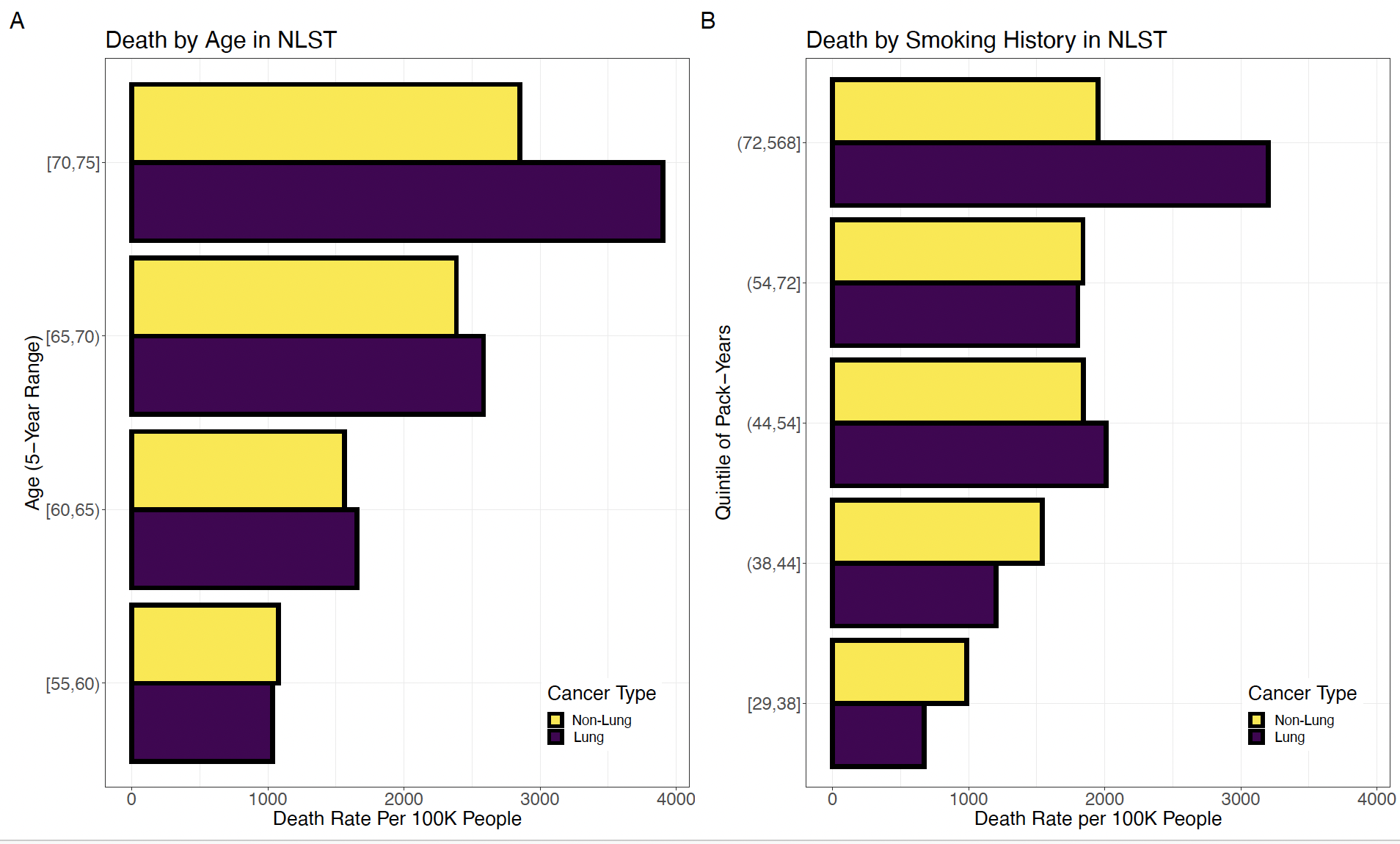
**

**Figure S5. Cancer-specific mortality rates (per 100,000 persons) from lung, other smoking-related, and non-smoking related cancers diagnosed during follow-up after randomization in the National Lung Screening Trial (NLST) study population stratified by A) age at enrollment or B) cigarette smoking pack-years at enrollment.**

**
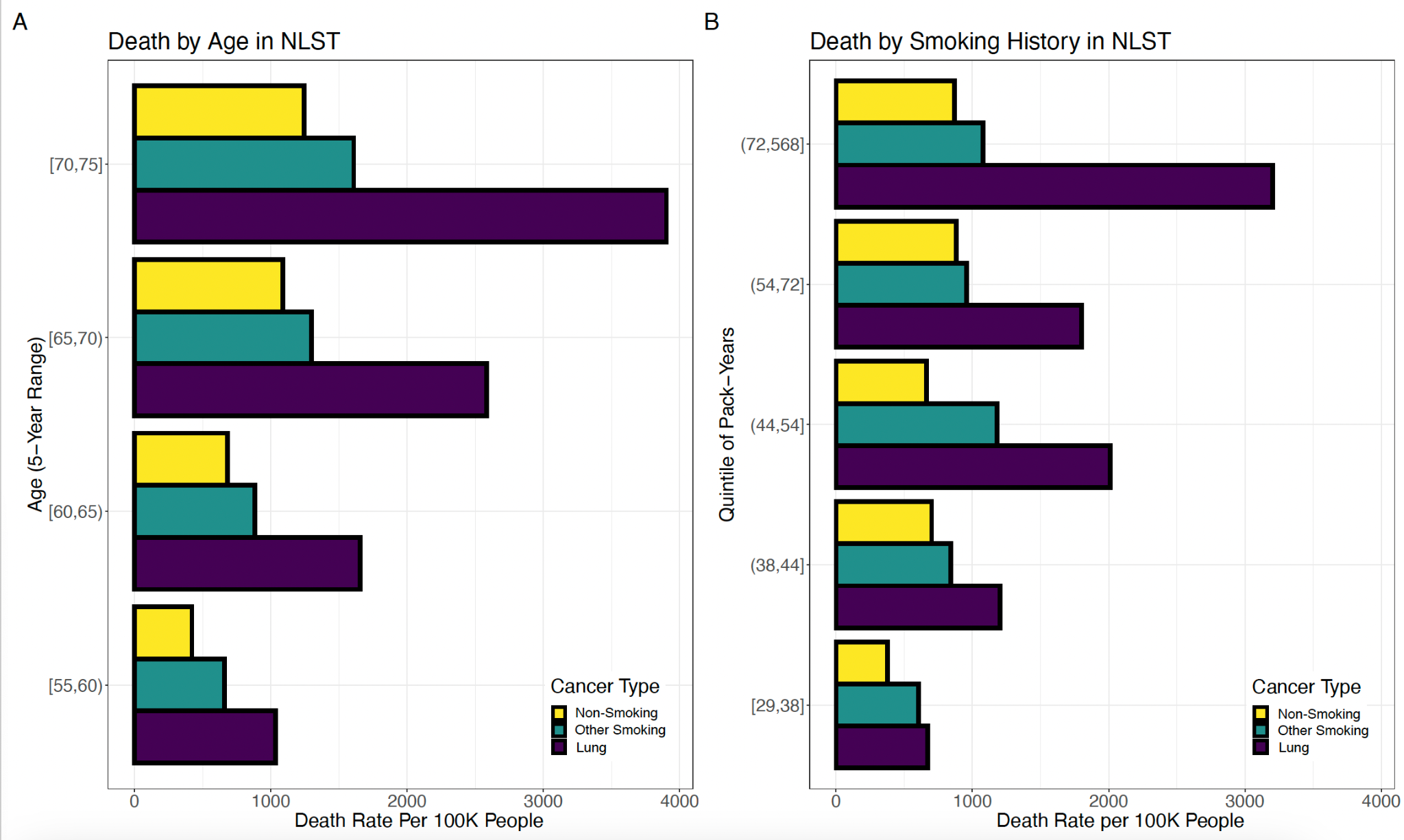
**

**Table S1. National Lung Screening Trial (NLST) subjects excluded from analysis.**

| **Category** | **n** |
| --- | --- |
| Individuals in initial dataset | 53,452 |
| Removed for ineligibility (n = 71 computed tomography within 18 months of enrollment, 29 participant in another cancer screening or prevention trial, 24 cancer within past 5 years, 23 non-smoker or quit > 15 years ago, 17 recent antibiotics use, 13 insufficient pack-years, 12 outside age range, 8 portion of lung removed, 2 metallic implants, 2 previous lung cancer, 1 home oxygen, 1 physical impairment to screening, 1 unexplained weight loss or hemoptysis) | 204 |
| Removed for invalid cancer status code | 11 |
| Removed for missing or invalid cancer diagnostic days | 4 |
| Removed for missing cancer behavior code | 2 |
| Removed for death 2 or more days before cancer diagnosis | 2 |
| Individuals in analytic dataset | 53,229 |
